# Evaluation of Pre-Transfusion Crossmatch Test Using Microscanner C3

**DOI:** 10.3390/diagnostics14121231

**Published:** 2024-06-12

**Authors:** Insu Park, Woong Sik Jang, Chae Seung Lim, Jeeyong Kim

**Affiliations:** 1 BK21 Graduate Program, Department of Biomedical Sciences, College of Medicine, Korea University, Seoul 02841, Republic of Korea; in2269@korea.ac.kr; 2 Departments of Emergency Medicine, College of Medicine, Korea University Guro Hospital, Seoul 08308, Republic of Korea; 3 Departments of Laboratory Medicine, College of Medicine, Korea University, Seoul 02841, Republic of Korea; 4 Departments of Laboratory Medicine, College of Medicine, Korea University Guro Hospital, Seoul 08308, Republic of Korea; 5 Departments of Laboratory Medicine, College of Medicine, Korea University Ansan Hospital, Ansan-si 15355, Republic of Korea

**Keywords:** crossmatch, transfusion, pre-transfusion, microscanner, blood bank

## Abstract

A pre-transfusion crossmatch test is crucial for ensuring safe blood transfusions by identifying the compatibility between donor and recipient blood samples. Conventional tube methods for crossmatching have limitations, including subjectivity in result interpretation and the potential for human error. In this study, we evaluated the diagnostic performance of a new crossmatch test using Microscanner C3, which can overcome these shortcomings. The crossmatch test results using the method were obtained in 323 clinical samples. The sensitivity, specificity, positive predictive value, negative predictive value, and concordance rate of the crossmatch test using Microscanner C3 were 98.20%, 100.00%, 100.00%, 98.11%, and 99.07%, respectively. The diagnostic performance of the new system offers a promising alternative to conventional tube methods for pre-transfusion crossmatch testing. Microscanner C3 could also increase the automation, standardization, and accuracy of crossmatch tests. The crossmatch test using Microscanner C3 is thought to increase the efficiency and reliability in identifying blood samples suitable for transfusion, thereby improving patient safety and optimizing the use of blood products in clinical settings.

## 1. Introduction

Healthcare professionals perform various pre-transfusion tests to transfuse the appropriate blood to patients. Clinically significant transfusion reactions typically manifest within the first 15 min of transfusion initiation, emphasizing the need for vigilant patient observation during this initial period. Prior to transfusion, measurements of the patient’s temperature, pulse, and respiratory rate are taken. Additionally, vital signs are assessed 15 min after the initiation of transfusion, and if any abnormalities are not noted, the transfusion is continued [1,2]. However, due to the potential life-threatening consequences of blood transfusion with an incompatible blood type resulting from incorrect sample collection, ensuring safe transfusion is of utmost importance. Acute hemolytic transfusion reactions, which can lead to severe outcomes, including death, must be diligently prevented. Differentiating between intravascular hemolysis caused by a major ABO mismatch transfusion and extravascular hemolysis induced by clinically significant alloantibody reactions is crucial. Notably, intravascular hemolysis presents with severe symptoms [2,3,4].

To confirm proper blood sampling from transfusion recipients, ABO and Rh blood typing and crossmatch tests must be conducted for each pre-transfusion sample [5]. Other blood typing is also performed to verify the blood product intended for transfusion. In clinical practice, ABO and Rh blood type tests are generally performed on patients before transfusion, and cross-matching, unexpected antibody screening, and identification tests can be additionally performed to ensure a safe transfusion [6]. The primary objective of crossmatch test is to detect anti-A, anti-B, and other antibodies in the transfusion recipient against the red blood cells (RBC) intended for transfusion [7]. This aims to prevent acute hemolytic transfusion reactions due to ABO blood type incompatibility and to mitigate acute hemolytic transfusion reactions resulting from unexpected antibodies in the transfusion blood reacting with corresponding antigens [8].

When no agglutination or hemolysis of RBCs in a crossmatch test is observed, it is considered a suitable blood and can be transfused. However, if agglutination or hemolysis is observed, this indicates a state of incompatibility, which means that the recipient has unexpected antibodies to the antigen of the tested RBC product. Crossmatch tests should be performed repeatedly until an RBC product without the corresponding antigen for the unexpected antibody is found [9,10]. The RBCs used in antibody screening tests include 2–3 blood cell panels that contain a wide range of clinically important blood types. O-type cells are used to detect reactions with unexpected antibodies rather than those against anti-A and anti-B. When an each of screening blood cell panel reacts with the recipient’s serum, it is judged positive if aggregation or hemolysis is observed. This indicates that antibodies against antigens contained in the positive panel of RBCs are present in the recipient [11,12].

If a crossmatch test or unexpected antibody screening test is positive, an unexpected antibody identification test should be performed to identify the type of unexpected antibody. Patients with previously identified antibodies should be transfused with an RBC product devoid of the antigen associated with the previous antibody. RBC products without relevant antigens should be transfused to these patients, even if unexpected antibodies are not currently detected [13,14]. This precaution is necessary to prevent delayed hemolytic transfusion reactions induced by a rapid increase in antibodies due to the anamnestic response when RBCs with specific antigens are transfused [15,16].

Before transfusion, medical staff must perform ABO blood type, Rh blood type, and crossmatch testing to ensure that the transfusion process is safe. If the antibody screening test is negative and there is no history of antibody detection, only a crossmatch test using the room temperature saline method can be performed to confirm the compatibility of the ABO and Rh blood type. If the antibody screening test is positive, a complete crossmatch, including the antiglobulin phase, must be performed to select alloantigen-negative blood [17,18].

In clinical laboratories, pre-transfusion test results including blood typing, crossmatch, unexpected antibody screening, and identification testing are interpreted by observing the presence of RBC agglutination [2,19]. The classical tube method offers the advantage of low-cost testing, because RBC aggregation can be observed and interpreted with the naked eye. However, it has the disadvantage of being labor-intensive and the interpretation of results may vary depending on the tester’s expertise in assessing RBC agglutination [20,21]. To compensate for the shortcomings of the test tube method and to standardize the results of the RBC agglutination reaction, microcolumn assays using a gel card have been developed. The microcolumn assay involves inserting RBCs that induce an agglutination reaction into a microtube contained in a gel, fixing the aggregates through centrifugation, and reading them [22,23]. The microcolumn assay is a more sensitive method than the classical tube method for detecting RBC alloantibodies. Large, automated equipment that can perform microcolumn assays has been developed and is being used in clinical laboratories with blood banks such as university hospitals and general hospitals [24,25].

However, microcolumn assays and classical tube methods, widely used in clinical practice, do not provide quantitative results that accurately reflect the level of RBC aggregation. When microcolumn assays use large, automated equipment for rapid testing, skilled professionals are required. Additionally, the size and cost of large equipment may make it difficult to use in clinical laboratories where space and finances are limited.

Pre-transfusion crossmatch testing between the donor and the recipient is an essential and critical step of the entire transfusion process [26,27]. Conventional crossmatch testing has been the mainstay of transfusion medicine for decades, but the standard tube method has many limitations [28]. One of the major drawbacks of traditional crossmatch tests using the tube method is the subjective nature of result interpretation. The visual assessment of agglutination patterns relies on the expertise and experience of laboratory technician, which can introduce variability and potential errors in result interpretation [29]. Conventional crossmatch testing often involves manual steps, including preparation of reagents, mixing of samples, and visual assessment of agglutination reactions. This labor-intensive process can be time-consuming and prone to human error, particularly in high-throughput laboratory settings where large numbers of samples need to be processed efficiently [30]. Conventional crossmatch testing methods may not reliably detect low-incidence antigens or antibodies, which could increase the risk of transfusion reactions in certain patient populations [31]. Without comprehensive antigen profiling, there is a potential for mismatched transfusions in individuals with rare blood types or alloantibodies. In addition, traditional tube methods do not provide quantitative data on the degree of agglutination, which can limit the ability to objectively assess compatibility between donor and recipient blood samples. Without quantitative analysis, it may be challenging to differentiate between weak or equivocal reactions and true incompatibilities. Despite these stringent quality control measures, traditional crossmatch testing methods remain vulnerable to human error, including sample labeling errors, transcription errors, and misinterpretation of results. These errors can have serious consequences, including transfusion of incompatible blood products and subsequent adverse patient outcomes. To overcome these shortcomings and improve test quality and result reproducibility, new methods such as solid-phase red blood cell adhesion assay (SPRCA) and red blood cell magnetization (EMT) are being attempted [32,33]. However, these methods are not widely used in clinical practice.

To overcome these shortcomings of existing methods, a new pretransfusion testing system using the Microscanner C3 (Biozentech, Seoul, Republic of Korea) has been developed. The Microscanner C3 is a microchip-based automated image analysis instrument consisting of a 10× magnification digital microscope equipped with automated image scanning and analysis software. Samples are dispensed onto disposable plastic microchips and then inserted into a small testing device for analysis. The Microscanner C3 can capture three types of fluorescence images, and automatically analyzes them using analysis programs. Moreover, the Microscanner C3 has the advantage of being able to analyze various targets through analysis programs such as cell counting, dot plots, and histograms. 

In this study, we evaluated the performance of the pre-transfusion crossmatch test using a Microscanner C3. This study was conducted with the expectation that anyone could easily and quickly perform a crossmatch test by automatically analyzing the RBC aggregation rate using the Microscanner C3. The diagnostic performance of the new crossmatch test using the Microscanner C3 was evaluated through a comparative evaluation with the tube method as a standard method in clinical samples. 

## 2. Materials and Methods

### 2.1. Sample Collection and Study Design

In this study, 323 ethylenediaminetetraacetic acid (EDTA) whole blood samples used in the pre-transfusion crossmatch test were obtained from patients who visited the Korea University Guro Hospital from October 2023 to June 2024. The collected samples were processed with anticoagulants contained in the EDTA tubes and centrifuged at 2000 rpm for 5 min to separate the serum and cell layers. Immediately upon retrieval, the upper layer (serum) was extracted and transferred to microtubes, then stored at −25 °C until just before the experiments, where they were thawed for use.

**Figure 1:**
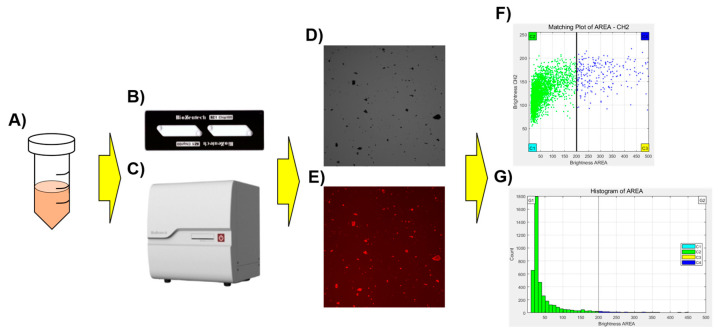
Schematic diagram of the Microscanner C3 for crossmatch testing. The stained sample (**A**) was dispensed onto the microchip (**B**), left for 3 min, and then the prepared chip was placed into Microscanner C3 (**C**) for analysis. Scatterplots (**F**) and histograms (**G**) for the captured optical image (**D**), and red fluorescence image (**E**) were generated using a clustering program built into the Microscanner C3.

All results obtained by a crossmatch test using Microscanner C3 were compared with those obtained by the conventional tube method as a standard method. This study was approved by the Medical Ethics Committee of Korea University Guro Hospital (2019GR0055) (Figure 1).

### 2.2. Microscanner C3

In this study, blood was administered to a microchip and the compatibility of the blood before transfusion was determined by automatically analyzing the RBC images contained in the microchip using Microscanner C3. 

The optical system of Microscanner C3 consists of a bright field and three fluorescent light-emitting diode (LED) channels. Images of various specimens and fluorescently stained samples can be obtained through a complementary metal oxide semiconductor (CMOS) camera. Images can be acquired using the autofocus function and do not require manual manipulation. In this study, bright field (BF) images were obtained using green light (530 ± 20 nm) without an emission filter. Red fluorescence images (Red Field, RF) were obtained under green light (530 ± 20 nm) using an emission filter (605 ± 27.5 nm). 

The BZ-1 chip, a disposable plastic chip (Biozentech, Seoul, Republic of Korea) used in the experiment, is made of polymethyl methacrylate (PMMA), a plastic optical material, and had dimensions of 25 (W) × 75 (D) × 1.7 (H) mm and contains two microfluidic channels with a depth of 100 µm. The use of disposable plastic microchips eliminates the need for warm-up or washing processes before and after use the equipment. Therefore, no contamination or carryover occurs during handling of specimens. Each channel can hold a sample volume of 20 μL, and the loading time for each channel ranges from 3 to 5 s. 

**Figure 2:**
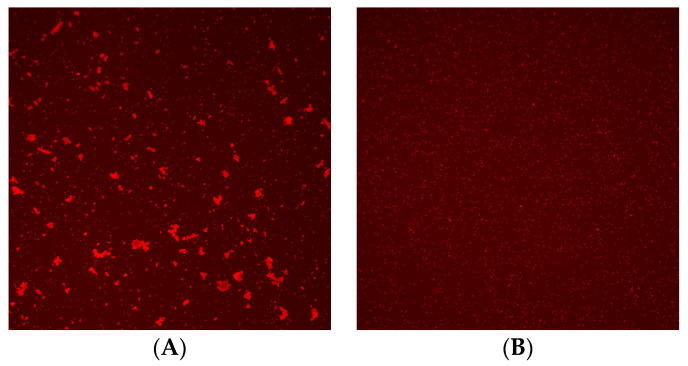
Image of crossmatch test using Microscanner C3. (**A**) Positive result and (**B**) negative result.

For image analysis, 20 μL of RBC aggregates stained with BD Pharmingen™ PE Mouse Anti-Human CD235a (BD Biosciences, Franklin Lake, NJ, USA) was dispensed onto the BZ-1 chip. BF and RF images of the BZ-1 chip were captured using an image analysis program built into the Microscanner C3. The images of RBCs were counted and output as a dot plot and histogram were obtained. The Y-axis represents the fluorescence brightness of RBCs and aggregates. In the histogram, the X-axis represents the area of RBCs and aggregates, and the Y-axis represents the frequency (Figure 2).

### 2.3. Crossmatch Test Using Microscanner C3

For comparison with standard methods using clinical samples, RBC agglutination tests were performed using a Microscanner C3. In total, 12.5 μL of 3% Screening Blood red cell (ID-DiaCell I & II, Bio-Rad, Hercules, CA, USA) was dispensed and 25 μL of patient serum was mixed with uniform vortexing at intensity 4 for 5 s into a 5 mL tube. This was vortexed again at intensity 4 for 3 s. After standing for 1 min, the mixture was centrifuged at 3000 rpm for 1 min to determine the degree of aggregation. It was then incubated at room temperature for 15 min and centrifuged at 3000 rpm for 1 min in the saline phase. In albumin stage, 12.5 μL of Serological Albumin 22% (Lorne Laboratories Limited, Reading, UK) was dispensed with vortexing intensity 4 for 3 s, incubated at 37℃ in a heat-block for 30 min, and then centrifuged at 3000 rpm for 1 min. In the anti-human globulin (AHG) stage, 500 μL of saline was dispensed and vortexed at intensity 4 for 5 s, and then it was centrifuged at 3000 rpm for 1 min, washed a total of 3 times and removing all supernatants. After dispensing 25 μL of polyspecific anti-human globulin (Merck Millipore, Darmstadt, Germany) with vortexing intensity 4 for 3 s, it was centrifuged at 3000 rpm for 1 min. After dispensing and resuspending 500 μL of phosphate-buffer saline (PBS), samples for experiments were prepared by vortexing at intensity 4 for 2 s. After dispensing 25 μL of the experimental sample into an opaque EP tube, 2 μL of CD235a-PE was added and vortexed with intensity 4 for 2 s, and then stored in the dark for 4 min. 

After gently resuspending, 20 μL of the stained sample was dispensed onto the BZ-1 chip and allowed to stand for 3 min. To capture Microscanner C3 10× BF/RF images, the BZ-1 was set to the initial focus RF, and then 15 fields were captured and analyzed with an AF range 200 and an AF step of 20. Microscanner C3 40× images were captured and analyzed for a total of 20 fields, including 10 fields for automatic capture and 10 fields for manual capture. 

**Figure 3:**
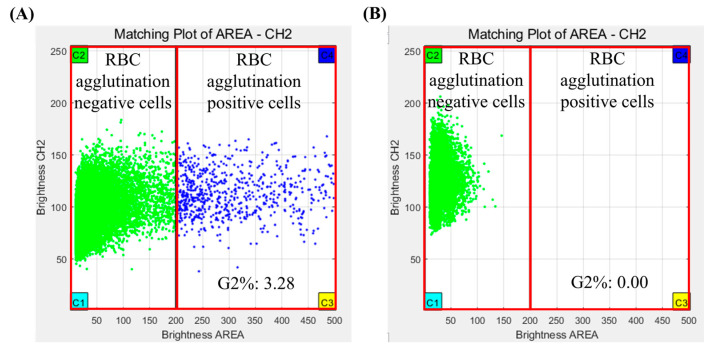
Crossmatch test results of Microscanner C3 by clustering analysis program. (**A**) Positive result and (**B**) negative result.

The results of the crossmatch test were interpreted using analysis software for Microscanner C3. The analysis software used fluorescence measurements repeatedly for all RBCs using a Microscanner C3 ver 1.0. Agglutination of RBCs was determined by measuring their fluorescence intensity and size of RBCs. The crossmatch test result was determined by G2%, which is the ratio of the number of agglutinated RBCs/total number of RBCs measured in captured images. If the G2% was more than 0.02, the crossmatch test result was judged positive, and if the G2% was less than 0.02, the crossmatch test result was judged negative (Figure 3).

### 2.4. Tube Method

In a 5 mL tube, 50 μL of 3% Screening Blood red cell (ID-DiaCell I & II) and 100 μL of patient sample were mixed by vortexing for 10 s. The tube was incubated at room temperature for 15 min and then centrifuged at 3000 rpm for 1 min. This test method was performed according to the reagent instructions, and the results were interpreted based on the degree of agglutination from negative to 4+ (positive). In the albumin stage, 50 μL of Serological Albumin 22% was dispensed with vortexing intensity 4 for 3 s, incubated at 37 °C in a heat-block for 30 min, then centrifuged at 3000 rpm for 1 min. In the anti-human globulin (AHG) stage, 1000 μL of saline was dispensed with vortexing intensity 4 for 5 s, then it was centrifuged at 3000 rpm for 1 min, and washed a total of 3 times, and supernatants were removed. After dispensing 100 μL of polyspecific anti-human globulin with vortexing intensity 4 for 3 s, it was centrifuged at 3000 rpm for 1 min. Each tube was independently interpreted, graded, and recorded by two examiners using an illuminated coherence viewer. 

### 2.5. Statistical Analysis

Test results obtained using Microscanner C3 were compared with those obtained using the tube method as a standard method for the pre-transfusion crossmatch test. The diagnostic performance of the crossmatch test using a Microscanner C3 was evaluated for clinical sensitivity, specificity, positive predictive value (PPV), negative predictive value (NPV), and concordance rate. The agreement rate between the new system and the existing tube method was calculated using inter-rater agreement statistics (Kappa). Statistical significance was set at *p* < 0.05. All statistical analyses were performed using SPSS for Windows (version 22.0; IBM Corporation, Armonk, NY, USA) and MedCalc version 22.016 (MedCalc software Ltd., Ostend, Belgium).

## 3. Results

Using a total of 323 clinical specimens, pre-transfusion crossmatch testing was performed using the tube method and a Microscanner C3, respectively. Of the 323 clinical samples, 167 samples were positive and 156 samples were negative for crossmatch test. Positive results of the crossmatch test using the Microscanner C3 and the tube method were observed in 164 and 167 samples, respectively. Negative results of the crossmatch test using the Microscanner C3 and tube method were observed in 159 and 156 samples, respectively.

The sensitivity of the crossmatch test using the Microscanner C3 and tube method was 98.20% and 100.00%, respectively. The specificity of the crossmatch test using the Microscanner C3 and tube method was 100.00% and 100.00%, respectively. The positive predictive value of the crossmatch test using the Microscanner C3 and tube method was 100.00% and 100.00%, respectively. The negative predictive value of the crossmatch test using the Microscanner C3 and the tube method was 98.11% and 100.00%, respectively. The concordance rate between the crossmatch test using the Microscanner C3 and the tube method was 99.07%, and Cohen’s Kappa coefficient was 0.981 (*p* < 0.001) (Table 1). 

The mean and standard deviation (SD) of G2% in positive samples for the crossmatch test was 1.1261 and 1.5405, respectively. The mean and standard deviation (SD) of G2% in negative samples for the crossmatch test was 0.0026 and 0.0029, respectively. There was a statistically significant difference in the average G2% between positive and negative samples (*p* = 0.000).

Unexpected antibody identification tests were performed on 167 crossmatch positive samples. In total, 10 unexpected antibodies including anti-C, anti-C+ anti-e, anti-E, anti-E+ anti-c, anti-Fy(b), anti-Le(a), anti-Le(a)+ anti-Le(b), anti-Le(b), anti-M, and anti-P1 were identified in 164 samples. All positive samples were classified according to the type of unexpected antibody (Table 2). 

Discordant results between the tube method and Microscanner C3 were found in three samples. In these samples, the G2% values of Microscanner C3 were 0.0000, 0.0000, and 0.0041, respectively. The corresponding unexpected antibodies were anti-Fy(b), anti-Fy(b), and anti-M, respectively (Table 3).

## 4. Discussion

This study presented a comprehensive evaluation of a new pre-transfusion crossmatch testing system, the Microscanner C3, designed to automate and standardize the crossmatch test process. The conventional tube method relies on visual observation for interpretation, which can lead to errors in assessing the degree of agglutination, especially when performed by less experienced technicians. In contrast, crossmatch testing using the Microscanner C3 provides automatic interpretation of agglutination levels, ensuring consistent results across different technicians and enhancing reproducibility. The classic tube method relies on visual observation of agglutination, which can lead to false positives or false negatives depending on the observer’s expertise. Notably, fine aggregation is particularly challenging to interpret, resulting in relatively low analytical sensitivity for detecting agglutination reactions [34,35]. The Korean Association of External Quality Assessment Service (KEQAS) conducts External Quality Assessments (EQA) of transfusion medicine in clinical laboratories in Korea. From 2019 to 2022, these EQAs resulted in 4681 unacceptable outcomes out of total 193,152 results. Of these, 4159 cases, or 88.8%, were attributed to incorrect interpretation of agglutination [36]. Out of a total of 58,726 results, 563 (0.96%) were incorrectly identified by the External Quality Assessment (EQA) conducted by the Austrian Association for Quality Assurance and Standardization of Medical and Diagnostic Tests. The error rate associated with the manual method was significantly higher than that of the automated method (1.04% vs. 0.42%) [37]. Additionally, while the conventional tube method allows for a semi-quantitative assessment of agglutination (−, ±, 1+, 2+, 3+, 4+), the Microscanner enables a quantitative evaluation of both the size and degree of agglutination. 

In this study, we compared the results of the crossmatch test performed using the Microscanner C3 with those obtained using the conventional tube method. The results indicated high sensitivity (98.20%) and specificity (100.00%) of the Microscanner C3 compared to the tube method. The positive and negative predictive values were also favorable, suggesting that the Microscanner C3 effectively identified compatible and incompatible blood samples. The high concordance rate (99.07%) and Cohen’s Kappa coefficient (0.981) between the two methods indicates that the Microscanner C3 performs comparably well to the conventional tube method in detecting RBC agglutination and determining compatibility. 

The new crossmatch test using Microscanner C3 has several advantages compared to conventional methods, including automation, standardization, and quantitative analysis possibilities. By automating the process of sample dispensing, imaging, and analysis, the Microscanner C3 reduces the labor-intensive nature of conventional crossmatch testing and minimizes the potential for human error. The use of disposable plastic microchips eliminates the need for complex sample preparation and reduces the risk of contamination, making the system user-friendly and suitable for routine clinical use. The time and cost required for crossmatch testing using Microscanner are comparable to those of the conventional tube methods, exhibiting minimal difference. However, establishing new laboratory facilities or operating under resource constraints may pose challenges in securing proficient personnel for crossmatch testing. Conversely, the utilization of Microscanner C3, facilitating straightforward interpretation even for novices, can prove advantageous in pre-transfusion crossmatch testing. Especially in the case of emergency blood transfusions, where even inexperienced technicians can quickly and effectively assess blood compatibility for transfusion based on Microscanner results. This Microscanner C3 could improve efficiency and reliability in pre-transfusion testing workflows in resource-limited settings.

However, this new crossmatch test using Microscanner C3 showed false negative results in 3 samples out of a total of 323 clinical samples. Crossmatch testing using Microscanner C3 on 2 anti-Fy(b) positive samples showed a G2% of 0.0000 in both samples, indicating a false negative result. We re-evaluated the performance of anti-Fy(b) using more samples and analyzed the cause of the errors. In this study, it was found that the failure to detect aggregation of Fy(b) in the crossmatch test using Microscanner C3 was due to the fact that the volume of AHG was lower than the test protocol. Upon resolving this error, further experiments were conducted on three Fy(b)-positive samples. As a result, agglutination was observed in all three samples during crossmatch testing with the Microscanner C3.

Despite the many advantages of the new crossmatch test using Microscanner C3, this study has several limitations. This study was limited by the variety of alloantibodies and the number of samples containing them, resulting in insufficient statistical analysis based on alloantibody types. Furthermore, despite the ability to quantitatively analyze agglutination levels, the clinical significance of these findings was not fully elucidated. In addition, this study did not investigate other potential factors that could influence the accuracy of the crossmatch test, such as the presence of interfering substances in patient samples or variations in sample handling procedures. While this study demonstrates promising results regarding the performance of the Microscanner C3 for pre-transfusion crossmatch testing, its findings should be interpreted with caution due to the limited sample size, patient population, and comparison scope. Further research with larger and more diverse patient cohorts, as well as comparative studies with other testing methods, would provide a more comprehensive understanding of the Microscanner C3’s utility and limitations in clinical practice.

## 5. Conclusions

In conclusion, this study demonstrated the effectiveness of the Microscanner C3 as a pre-transfusion crossmatch testing system in clinical samples. The new crossmatch test using Microscanner C3 offers a promising alternative to conventional tube methods because of its ability to automate and standardize the testing process, reduce turnaround time, and improve accuracy. The new crossmatch test using Microscanner C3 is believed to be an accurate test that ensures safe and compatible blood transfusions through automation and standardization. By enhancing the efficiency and reliability of pre-transfusion testing, the Microscanner C3 has the potential to enhance patient safety and optimize blood product utilization in clinical settings. Further research and validation studies are warranted to confirm the findings and assess the long-term impact of implementing the Microscanner C3 in clinical transfusion practice.

## Figures and Tables

**Table 1:** Results of crossmatch test using tube method and Microscanner C3.

Crossmatch Test	Tube Method		Microscanner C3	
	Positive	Negative	Positive	Negative
Positive samples	167	0	164	3
Negative samples	0	156	0	156
Total	167	156	164	159

**Table 2:** The number of positive results according to unexpected antibody.

Unexpected Antibody	Tube Method	Microscanner C3
anti-C	2	2
anti-C + anti-e	48	48
anti-E	13	13
anti-E + anti-c	16	16
anti-Fy(b)	5	3
anti-Le(a)	28	28
anti-Le(a) + anti-Le(b)	23	23
anti-Le(b)	17	17
anti-M	11	10
anti-P1	4	4
Total	167	164

**Table 3:** Discordant results between the tube method and Microscanner C3.

Unexpected Antibody	Tube Method	Microscanner C3	
		Result	G2%
anti-Fy(b)	Positive	Negative	0.0000
anti-Fy(b)	Positive	Negative	0.0000
anti-M	Positive	Negative	0.0041

## Data Availability

The authors declare that all related data are available from the corresponding author upon reasonable request.
